# Estimation of Plate and Bowl Dimensions for Food Portion Size Assessment in a Wearable Sensor System

**DOI:** 10.1109/jsen.2023.3235956

**Published:** 2023-01-24

**Authors:** Viprav B. Raju, Delwar Hossain, Edward Sazonov

**Affiliations:** Department of Electrical and Computer Engineering, The University of Alabama, Tuscaloosa, AL 35401 USA.

**Keywords:** Dietary assessment, food imaging, food portion, food volume, portion size estimation, wearable sensors, wearable technology

## Abstract

Automatic food portion size estimation (FPSE) with minimal user burden is a challenging task. Most of the existing FPSE methods use fiducial markers and/or virtual models as dimensional references. An alternative approach is to estimate the dimensions of the eating containers prior to estimating the portion size. In this article, we propose a wearable sensor system (the automatic ingestion monitor integrated with a ranging sensor) and a related method for the estimation of dimensions of plates and bowls. The contributions of this study are: 1) the model eliminates the need for fiducial markers; 2) the camera system [automatic ingestion monitor version 2 (AIM-2)] is not restricted in terms of positioning relative to the food item; 3) our model accounts for radial lens distortion caused due to lens aberrations; 4) a ranging sensor directly gives the distance between the sensor and the eating surface; 5) the model is not restricted to circular plates; and 6) the proposed system implements a passive method that can be used for assessment of container dimensions with minimum user interaction. The error rates (mean ± std. dev) for dimension estimation were 2.01% ± 4.10% for plate widths/diameters, 2.75% ± 38.11% for bowl heights, and 4.58% ± 6.78% for bowl diameters.

## Graphical Abstract

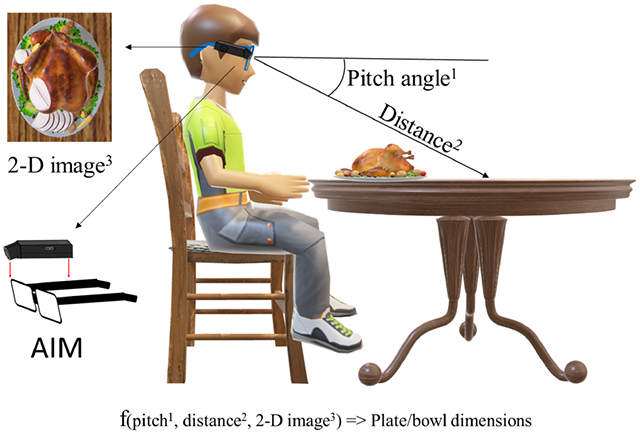

## I. Introduction

Reliable and accurate portion size estimation is challenging but essential for dietary assessment. Image-based dietary assessment has been one of the fastest growing areas of research in this milieu. Image-based assessment can be split into manual assessment and automatic assessment. Manual assessment can be done using digital food records [2] or by image-based 24-h recall/self-reporting that involve food atlases [3], [4], [5], [6], [7]. The image-based food records involve capturing meal images that are reviewed later by the participant or by a professional (nutritionist/clinician/researcher) to estimate the portion size. Digital food images are a useful tool for the quantification of food items and in portion size estimation [8], [9], [10]. Images of food leftovers are also captured in some studies, which improved the portion size accuracy [11]. Recall or self-reporting methods use food atlases. Food atlases are reference guides that are taken to present various portions representative of the range of portion sizes usually consumed. Either during or after data collection, participants are asked to report the food quantity consumed by selecting a particular image, a fraction/multiple of an image, or a combination of images [12].

The abovementioned manual methods are cumbersome, subject to memory (and therefore prone to error), and are not accurate compared to the much recent automatic assessment methods. A previous review [13] identified some of the existing image-based food portion size estimation (FPSE) methods that are automatic. It was seen that food portion size can be estimated automatically using food images captured during the meal [14], [15], [16], [17], [18]. However, automatic FPSE from food images is a challenging task since the two-dimensional (2-D) image lacks the three-dimensional (3-D) real-world information. There is a lack of reference to measure/gauge the size/volume of the food items. To tackle this problem, the dimensional reference is obtained by using a visual cue that must be present in a food picture. A few methods used virtual objects or objects that already exist in a typical food image to aid in FPSE. Some of the popular approaches included geometric models [19], VR-based referencing [20], circular object referencing [17], [21], and thumb-based referencing [22]. Shang et al. [23] used a structured light-based 3-D reconstruction approach to estimate food volume. Jia et al. [17] used the “*plate method*” for FPSE where the circular plates present in the image are used to determine location, orientation, and volume of the food items. The study, however, only considers circular plates.

A fiducial marker of known dimensions placed in the images can also be used as a point of reference [17], [22], [24], [25]. The type of reference used determines the complexity of setup. Some methods require the users to carry around the reference (checkerboards, blocks, and cards) and some require special dining setups, which increases user burden.

Another classification in image-based FPSE can be done based on the mode of image capture. Food image capture can either be *active* or *passive*. Active methods rely on the participant to capture the food image by a camera (such as a smartphone camera), typically, before and after an eating episode. The images are then processed using computer vision models to segment foods, recognize foods, estimate portion size/volume, and compute energy content [26], [27], [28]. Active methods provide detailed information such as meal timing, location, and duration of the eating episodes. However, these methods require the active participation of the users, which can be a burden. Some of the active methods that predict portion sizes require fiducial markers in the food image to assist manual review/computer algorithms [26], [29]. The placement of these markers combined with the active nature of image capture increases the user burden considerably.

One study presented a new *active* method for food volume estimation without using a fiducial marker. The method utilizes a special picture-taking strategy on a smartphone [1]. A mathematical model that uses the height and orientation of the smartphone was used to determine the real-world coordinates of the plane of the eating surface in the capture image. Bucher et al. [30] presented and tested a new virtual reality method for food volume estimation using the International Food Unit. This method, however, requires the user to place the smartphone on the eating surface while image capture and also needs additional user interaction in using the virtual International Food Unit.

Food images can also be acquired by a “passive” method using wearable devices that capture images continuously (both food and nonfood) without the active participation of the user [31], [32]. The passive methods minimize the burden of active capture using a wearable camera. However, FPSE methods that require fiducial markers cannot be easily integrated with the passive image capture since the user is not actively taking images and do not know when to place these markers.

The automatic ingestion monitor [33] is a wearable sensor system [automatic ingestion monitor version 2 (AIM-2)] that is mounted on a user’s eyeglass. The sensor consists of a combination of sensors for accurate detection of food intake and triggering of a wearable camera (*passive*). In this study, we integrate a time-of-flight (ToF) ranging sensor with AIM-2 and propose a novel method to determine container dimensions (bowls/plates). The method does not require fiducial markers. Once the size of the vessels is known, portion size can be estimated using the “plate method.” In this study, our objective is to measure the dimensions of plates and bowls.

The major contributions of the proposed work are: 1) the model eliminates the need for fiducial markers; 2) the camera system (AIM-2) is not restricted in terms of positioning relative to the food item; 3) our model accounts for radial lens distortion in caused due to lens aberrations; 4) a ranging (ToF) sensor directly gives the distance between the sensor and the eating surface; 5) the model is not restricted to circular plates; and 6) a passive method that can be used for assessment of container dimensions with minimum user interaction.

## II. Methods

### A. Equipment

In this study, a novel wearable sensor system (AIM-2 with a ToF ranging sensor) was used [33]. AIM-2 consists of a sensor module, which houses a miniature 5-Mpixel camera with 120° wide-angle gaze-aligned lens, a low-power 3-D accelerometer (ADXL362 from Analog Devices, Norwood, MA, USA), and a ToF ranging sensor (VL53L0X from STMicroelectronics). The sensor system is enclosed in a custom-designed 3-D printed enclosure. The ToF sensor is aligned with the camera axis.

**Fig. 1.**
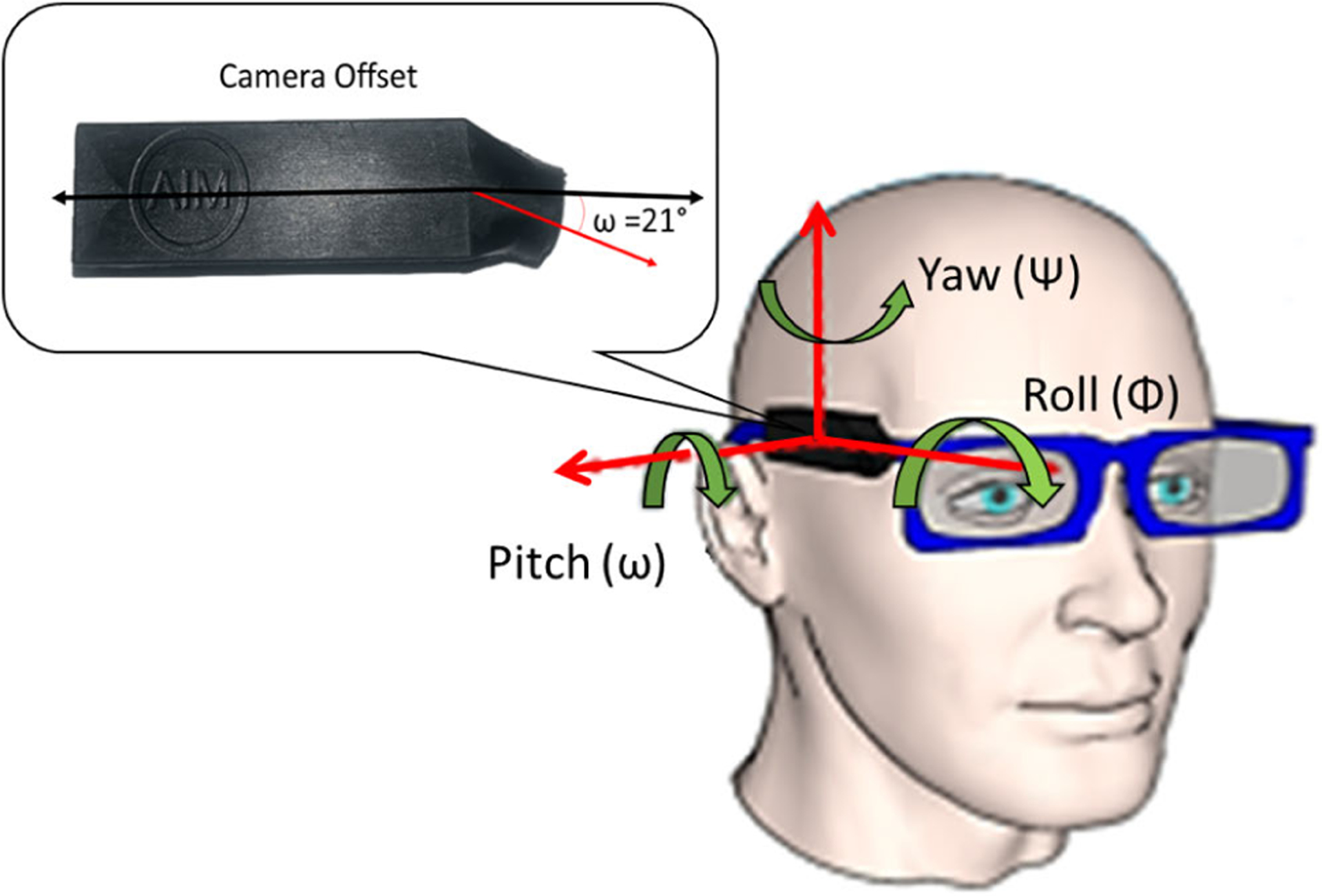
Rotational axes of the AIM sensor. Also depicted is the camera offset of 21° with respect to the axis of the eyeglass/AIM device.

The camera continuously captured images at a rate of one image per 10-s interval continuously throughout the day. Data from the accelerometer and ToF sensor were sampled at 128 Hz. All collected sensor signals and captured images were stored on an SD card and processed off-line in MATLAB (MathWorks Inc., Natick, MA, USA) for algorithm development and validation. The AIM enclosure is such that the camera and the ToF sensor are at an angle of 21° with respect to the accelerometer axis, as shown in Fig. 1. We will be using this offset (+21°) while calculating the pitch of the camera. The raw sensor data from the accelerometer were preprocessed before extracting the pitch angle. A high-pass filter with a cutoff frequency of 0.1 Hz was applied to remove the dc component from the accelerometer signal.

**Fig. 2.**
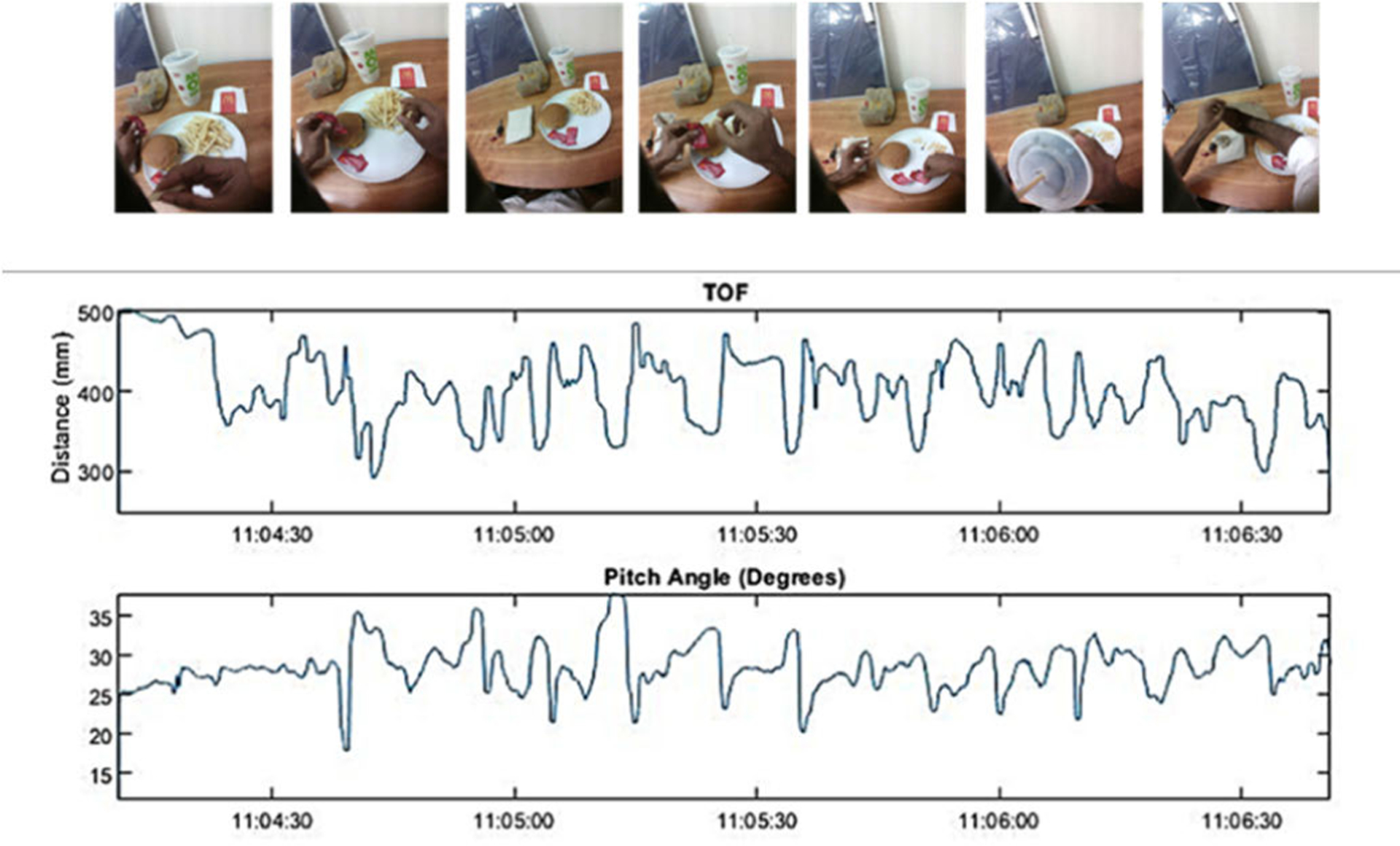
ToF distance and pitch angle as a function of time (*y*-axis: hh:mm:ss) for a sample meal where the AIM-2 was used.

The sensor pitch was calculated as in [33] and the device pitch is obtained by adding the offset (21°) to the sensor pitch. The distance readings are more straightforward, the raw values depicting the actual distances. Next, using the timestamp of an image, the respective pitch and distance readings were extracted. Fig. 2 shows the ToF distance readings and pitch plotted as a function of time for a sample meal.

### B. Geometric Model

The objective is to project the points in an image in the real-world coordinates. In this study, our primary concern is to measure the dimensions of the plate and bowls.

**Fig. 3.**
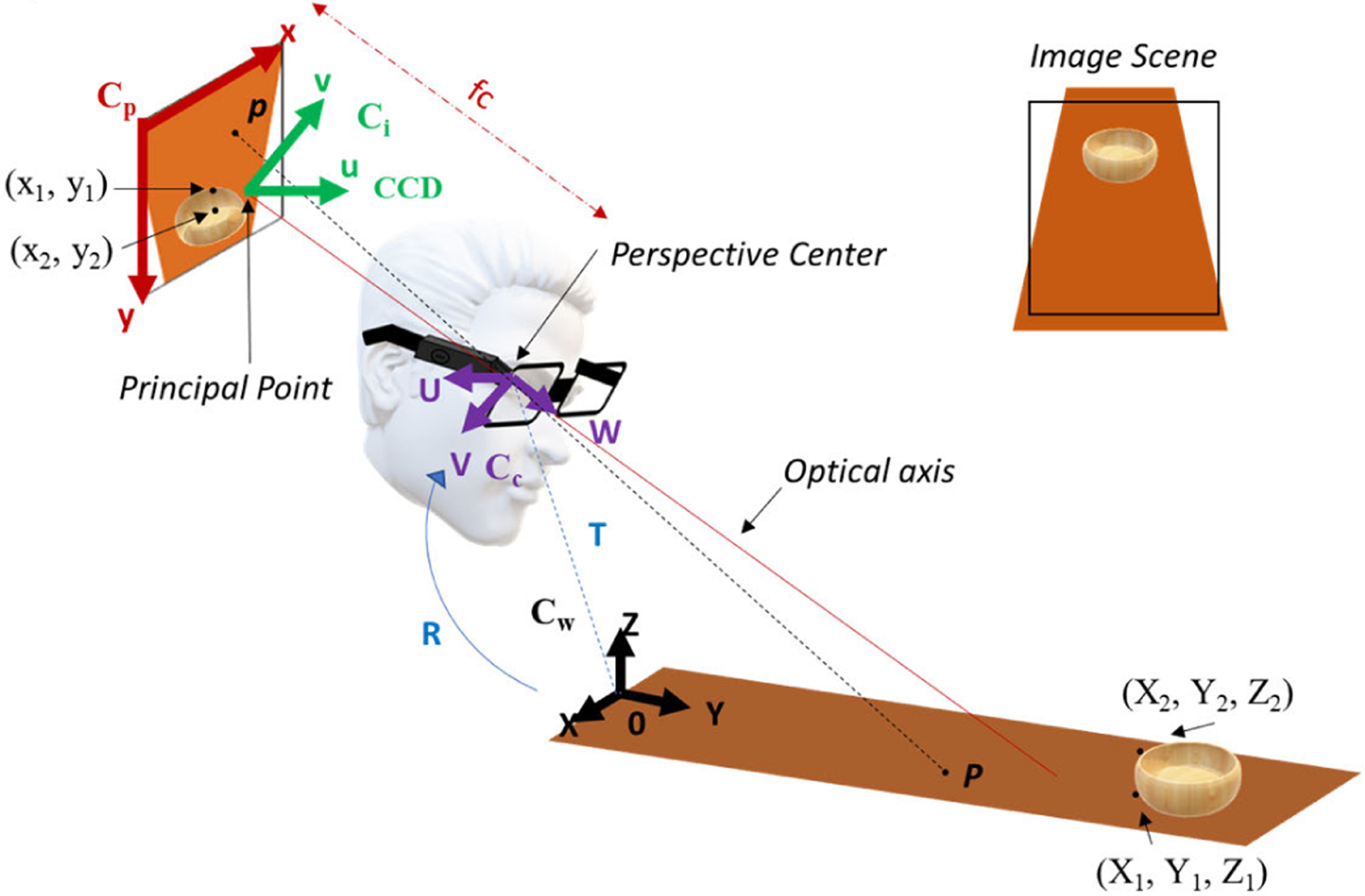
Model of the AIM imaging system.

Refer to Fig. 3. Let P be a point in the world, *C*_*w*_ be a world coordinate system, and (*X Y Z*)^t^ be the coordinates of P in *C*_*w*_. Define the camera coordinate system, *C*_*c*_, to have its *W*-axis parallel with the optical axis of the camera lens, its *U*-axis parallel with the *u*-axis of *C*_*i*_ (image coordinate plane), and origin located at the perspective center. Let (*U V W*)^t^ be the coordinates of P in *C*_*c*_. The coordinates (*U V W*)^t^ are related to (*X Y Z*)^t^ by a rigid body coordinate transformation

(1) $$\left(\begin{array}{l} U\\ V\\ W \end{array}\right)=R\;\left(\begin{array}{l} X\\ Y\\ Z \end{array}\right)+T$$

where *R* is a 3 × 3 rotation matrix and *T* is a 3 × 1 translation vector. *R* is dependent on three angles of rotation, namely, pitch (*ω*), roll (Φ), and yaw (ψ). The three angles for the AIM device are shown in Fig. 1.

The principal point is the intersection of the imaging plane with the optical axis. Let *f*_*c*_ be the focal length of the lens of the imaging system. Define the 2-D image coordinate system, *C*_*i*_, to be in the image plane with its origin located at the principal point, *u*-axis in the fast scan direction (horizontal rows of pixels on the sensor), and *v*-axis in the slow scan (vertical rows of pixels on the sensor) direction of the camera sensor. Fast scan indicates the pixel direction in which the sensor scans at a higher rate. Let *p* be the projection of P onto the image plane and let ${\left(\bar{\text{u}},\bar{v}\right)}^{\text{t}}$ be the coordinates of p in *C*_*i*_. The focal length (Table. I) of ${\left(\bar{\text{u}},\bar{v}\right)}^{\text{t}}$ is given by

(2) $$\left(\begin{array}{l} \bar{\text{u}}\\ \bar{v} \end{array}\right)=\frac{f_{c}}{W}\left(\begin{array}{l} U\\ V \end{array}\right).$$

Next, radial lens distortion is incorporated into the model in the following way. Let (*uv*)^t^ be the actual observed image point after being subject to lens distortion. Then, (*u v*)^t^ is related to ${\left(\bar{\text{u}},\bar{v}\right)}^{\text{t}}$ by

(3) $$\left(\begin{array}{l} u\\ v \end{array}\right)=\left(\begin{array}{c} \bar{\text{u}}\\ \bar{v} \end{array}\right)\left[1+K_{c}\left({\bar{\text{u}}}^{2}+{\bar{v}}^{2}\right)\right]$$

where *K*_*c*_ is a coefficient, which controls the amount of radial distortion.

Finally, it is necessary to model the image sampling performed by the camera sensor [charged coupled device (CCD)]. A camera sensor consists of a 2-D array of photosensors. Each photosensor converts incoming light into a digital signal by means of an analog-to-digital converter. To obtain color information, one “sensor pixel” is divided into a grid of photosensors, and different color filters are placed in front of these multiple photosensors. Each of these photosensors receives light through only one of the three filters: blue, red, and green. Combining these measurements gives one color triple: (red intensity, green intensity, and blue intensity). This is known as the Bayer filter. Therefore, the digital image coordinates are not the same as the pixel coordinates.

Let *C_p_* be the pixel coordinate system associated with the digital image. The pixel coordinates are related to the image coordinates by

(4) $$\left(\begin{array}{l} x\\ y \end{array}\right)=\left(\begin{array}{cc} s_{c}^{x} & K_{c}\\ 0 & s_{c}^{y} \end{array}\right)\left(\begin{array}{l} u\\ v \end{array}\right)+\left(\begin{array}{c} c_{c}^{x}\\ c_{c}^{y} \end{array}\right)$$

where $s_{c}^{x}$ and $s_{c}^{y}$ are scale factors (pixel/mm), $c_{c}^{x}$ and $c_{c}^{y}$ are the pixel coordinates of the principal point, and *K*_*c*_ is the distortion coefficient (pixel/mm)

(5) $$\left(\frac{x}{y}\right)=f\;\left(XYZ\right).$$

We are interested in the inverse function of (5) for the purposes of dimension estimation, i.e.,

(6) $$\left(\begin{array}{l} X\\ Y\\ Z \end{array}\right)=f^{-1}\;\left(xy\right).$$

With the known AIM sensor orientation, namely, the pitch angle of the sensor, provided by the inertial measurement unit, a right angle between the surface of the lens and the optical axis, and the projection relationship in (5), it can be shown that the inverse of function *f* in (6) exists for the tabletop, i.e.,

(7) $$\left(\begin{array}{l} X\\ Y\\ 0 \end{array}\right)=f^{-1}\;\left(xy\right).$$

Note that *Z* = 0 in (7) represents the plane equation of the tabletop.

Also, we assume that the roll (Φ) and yaw (ψ) of the sensor are zero. Therefore, the rotation matrix *R* is given by

(8) $$R=\left[\begin{array}{ccc} 1 & 0 & 0\\ 0 & \text{cos}\omega & -\text{sin}\omega \\ 0 & \text{sin}\omega & \text{cos}\omega \end{array}\right]\text{.}$$

From (4), the world coordinates of the tabletop are related to the pixel coordinates by

(9) $$\left(\begin{array}{l} u\\ v \end{array}\right)=\left(\begin{array}{cc} 1/s_{c}^{x} & 0\\ -Kc & 1/s_{c}^{y} \end{array}\right)+\left(\begin{array}{l} x-c_{c}^{x}\\ y-c_{c}^{y} \end{array}\right).$$

**Fig. 4.**
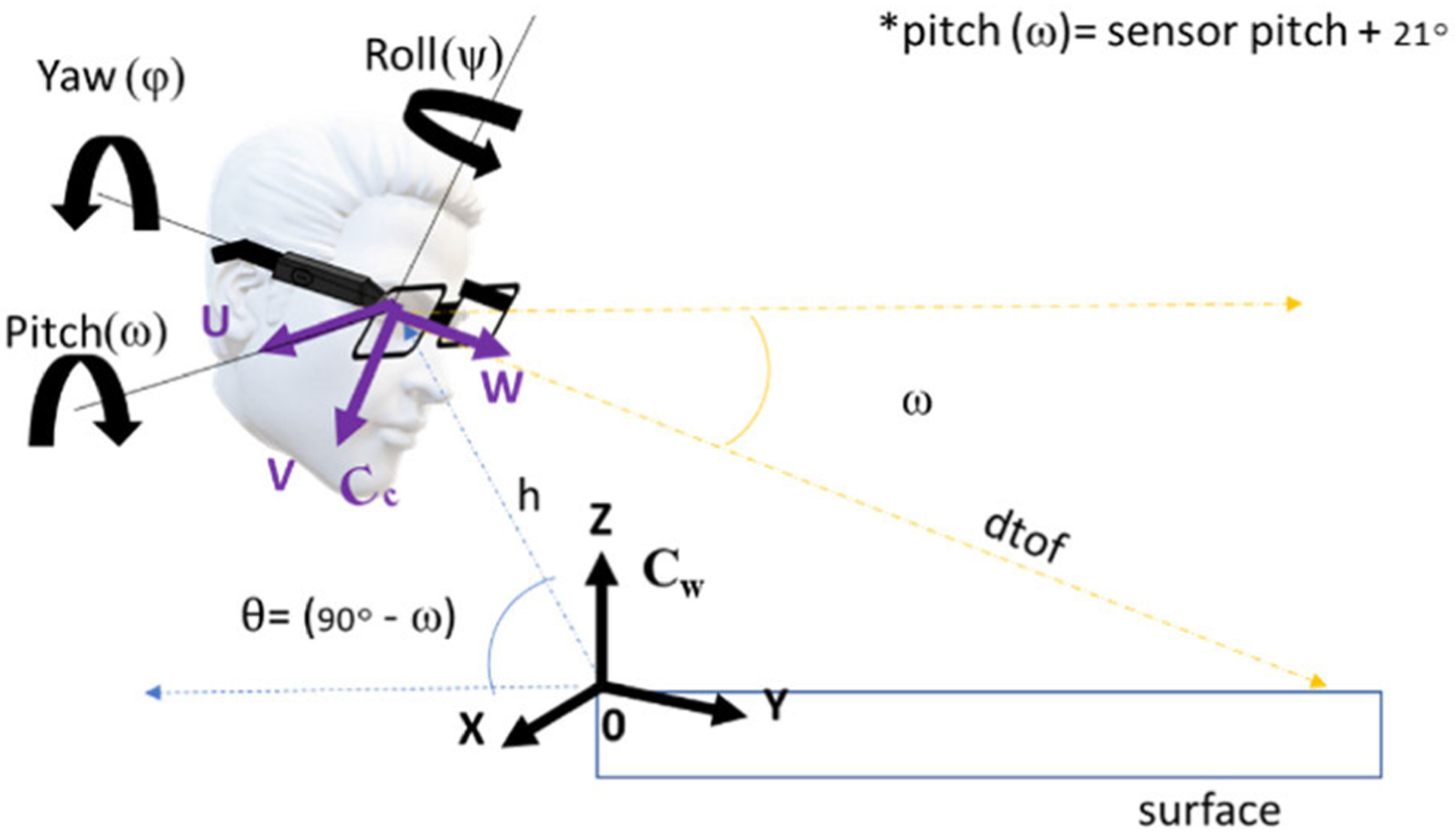
Projection of camera coordinates on to the world coordinates. Also, depict the calculation of pitch angle of the camera.

To calculate the translational and rotation matrices, we make use of the sensor pitch (*ω*) and distance readings from the AIM. The distance is obtained by the ToF sensor, and the sensor pitch is obtained by the accelerometer on the AIM device, as shown in Fig. 4. The camera on the AIM has an offset of 21°

(10) h=dtof×tan(ω)

where *d* tof is the distance from the ToF sensor, which is the distance between the AIM and the eating surface. As in [1], we obtain the following equation:

(11) $$W=\frac{h\cdot \text{sin}(\theta )}{\text{cos}(\theta )-\frac{v\cdot \text{sin}(\theta )}{f_{c}}}\text{.}$$

From (9), we obtain

(12) $$\left(\begin{array}{l} U\\ V \end{array}\right)=\frac{W}{f_{c}}\left(\begin{array}{l} u\\ v \end{array}\right).$$

Finally, we obtain the equation

(13) $$\left(\begin{array}{l} X\\ Y\\ 0 \end{array}\right)=R^{-1}\;\left(\left(\begin{array}{c} U\\ V\\ W \end{array}\right)-T\right)$$

where *T* = [0; −*h*; 0].

Equation (13) gives us the plane of the eating surface (*Z* = 0).

The study mainly focuses on obtaining the dimensions of two types of vessels, namely, plates and bowls. We assume plates to be flat and part of the plane *Z* = 0. The heights/depths of the plates are assumed to be negligible and approximated to zero. We measure the dimensions of the plate on this plane.

**Fig. 5.**
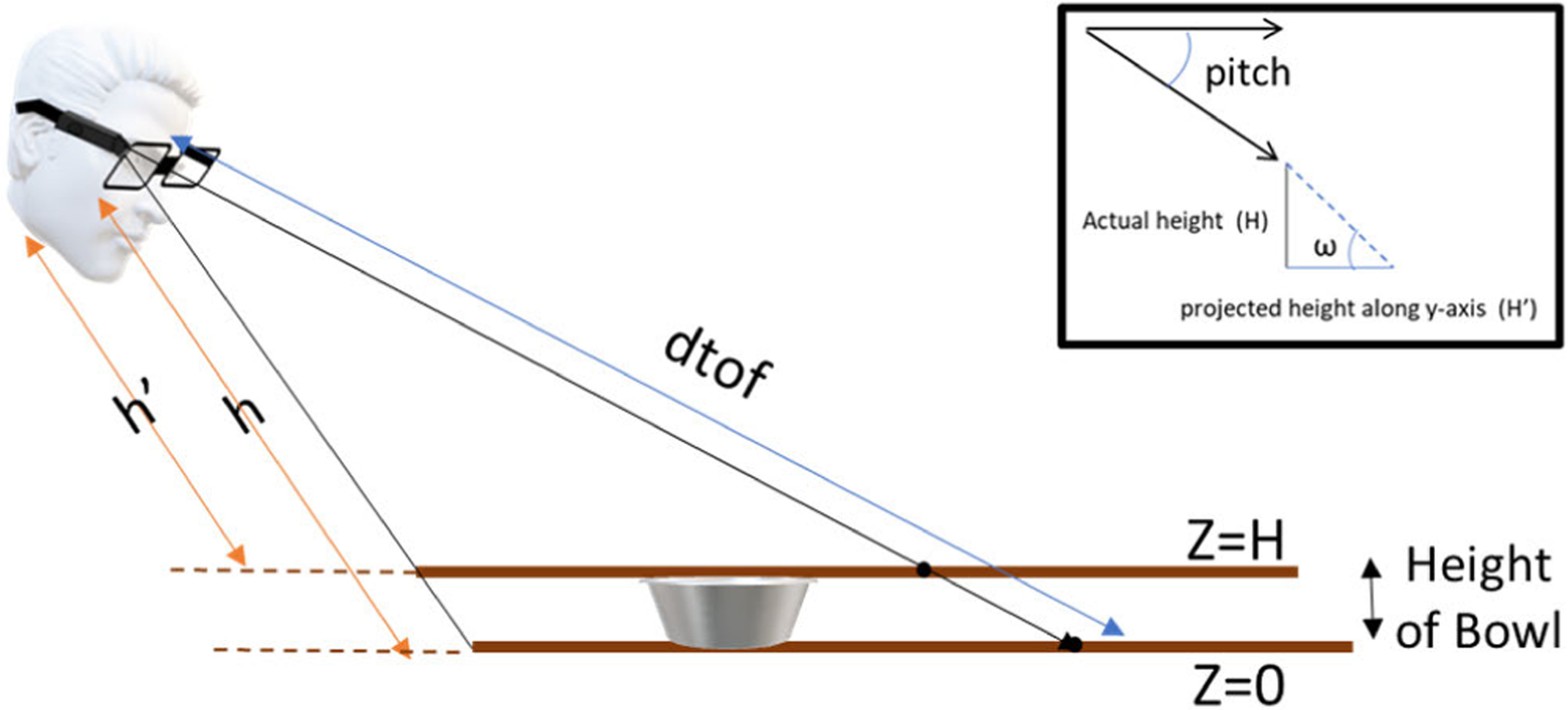
Projection *H*’ of height *H* on the *y*-axis. Calculating *h*’ for obtaining the plane equation for *Z* = *H*.

However, in the case of bowls, first, the height of the bowl is measured along the *y*-axis, as shown in Fig. 5. The height is just a projection on the *y*-axis and the true height is calculated as in (14). Here, the assumption is that the bowl sides are flat and not curved

(14) $$H=\text{tan}\;\left(\omega \right)\times H^{\prime }.$$

Once the height of the bowl (*H*) is calculated, the equation of the plane *Z* = *H* is obtained instead of *Z* = 0. Fig. 5 shows the changes in the parameters for obtaining the adjusted plane equation

(15) $$h^{\prime }=h-\left(H\times \text{sec}\;\left(\omega \right)\right)$$

where *h* is calculated as in (10).

Once *h*′ is obtained, this value is plugged into (11) followed by (12) and (13).

We then measure the dimensions of the mouth of the bowls similar to the dimensions of the plates. We assume bowl of the mouth to be a part of the plane *Z* = *H*. The radius of the mouth of the bowl is then measured on this plane along the *x*- and *y*-axes.

### C. Data

**Fig. 6.**
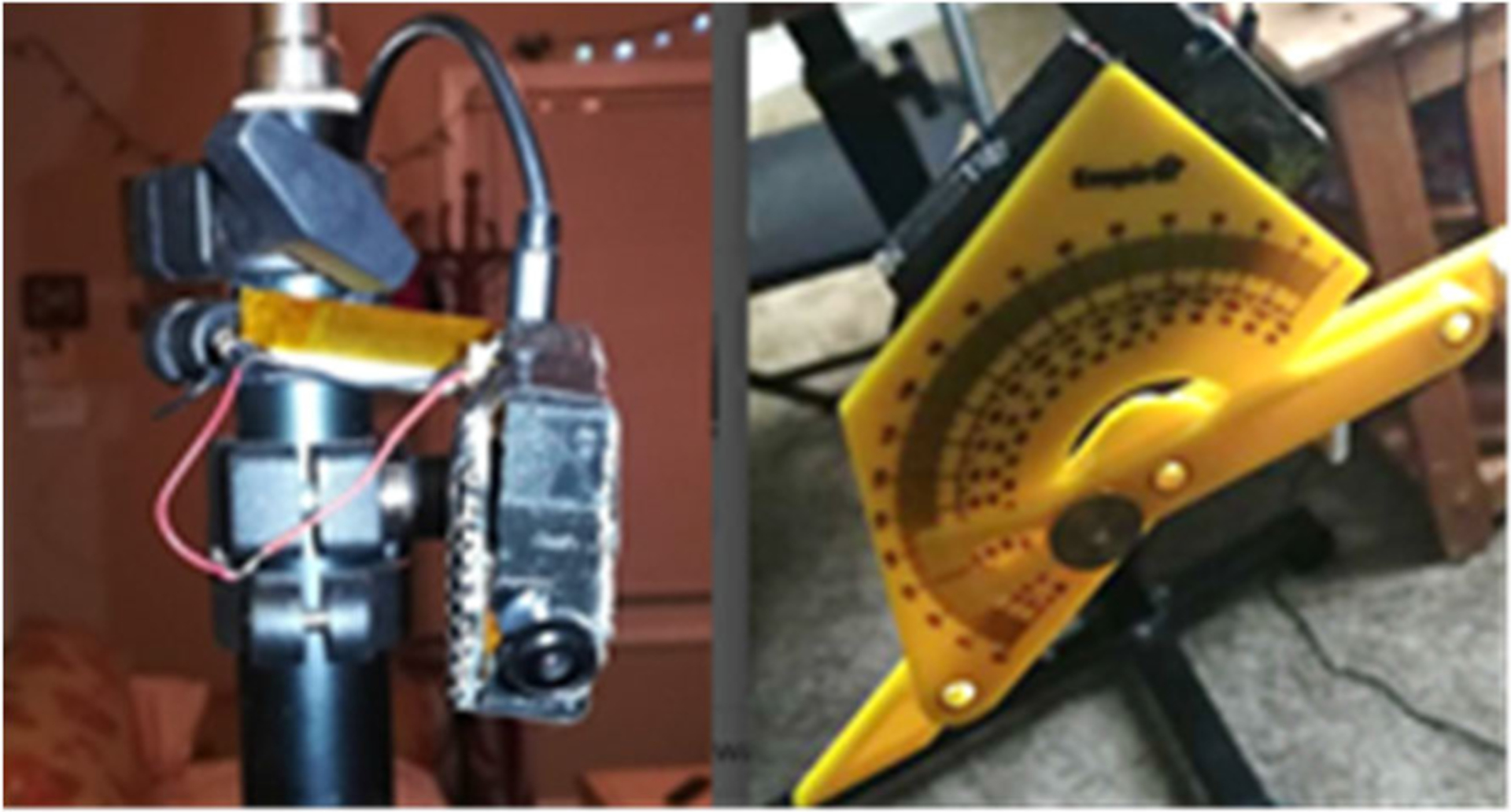
Left: test bench with the AIM attached to the tripod. Right: protractor used to measure the pitch angle of the sensor system.

The AIM sensor system was mounted on a test bench to collect data. The test bench consisted of a tripod and a protractor for angle measurement (see Fig. 6). The AIM device was placed on a tripod in front of a table at three pitch angles (40°, 55°, and 70°) with respect to the parallel to the ground, at three different heights from the table surface (20, 35, and 50 cm). The angles were measured using the protractor fixed to the side of the sensor aligned with the camera (as shown in Fig. 6). The protractor was also calibrated to test for errors in the pitch angle measurement. The calibration was done in increments of 10° from 0° to 70°. The error in measurement was (mean ± std. dev) −2.43°±1.36°. The roll and yaw of the cameras were approximately set to be 0 for experimentation. Also, the roll and yaw for the AIM are assumed to be 0 when a person is eating.

**Fig. 7.**
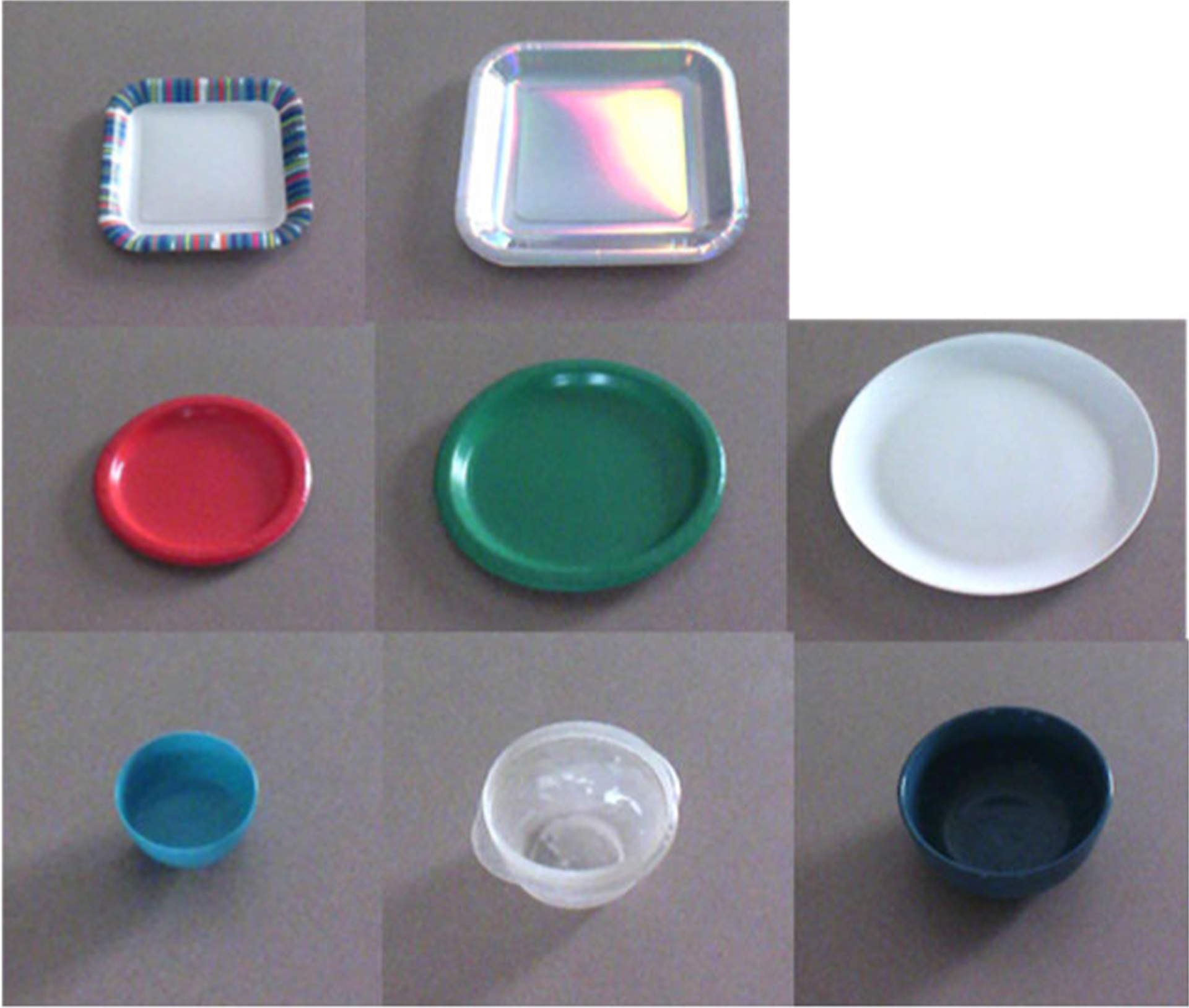
Test dataset: plates and bowls of varying sizes.

Nine sets of data collected at a combination of three heights and three pitch angles were used for testing (see Fig. 7). A set of eight objects, three circular plates (diameter: small 18 cm, medium 22 cm, and large 26 cm), two square plates (side: small 18 cm and medium 23 cm), and three circular bowls, were used as objects of interest.

**Fig. 8.**
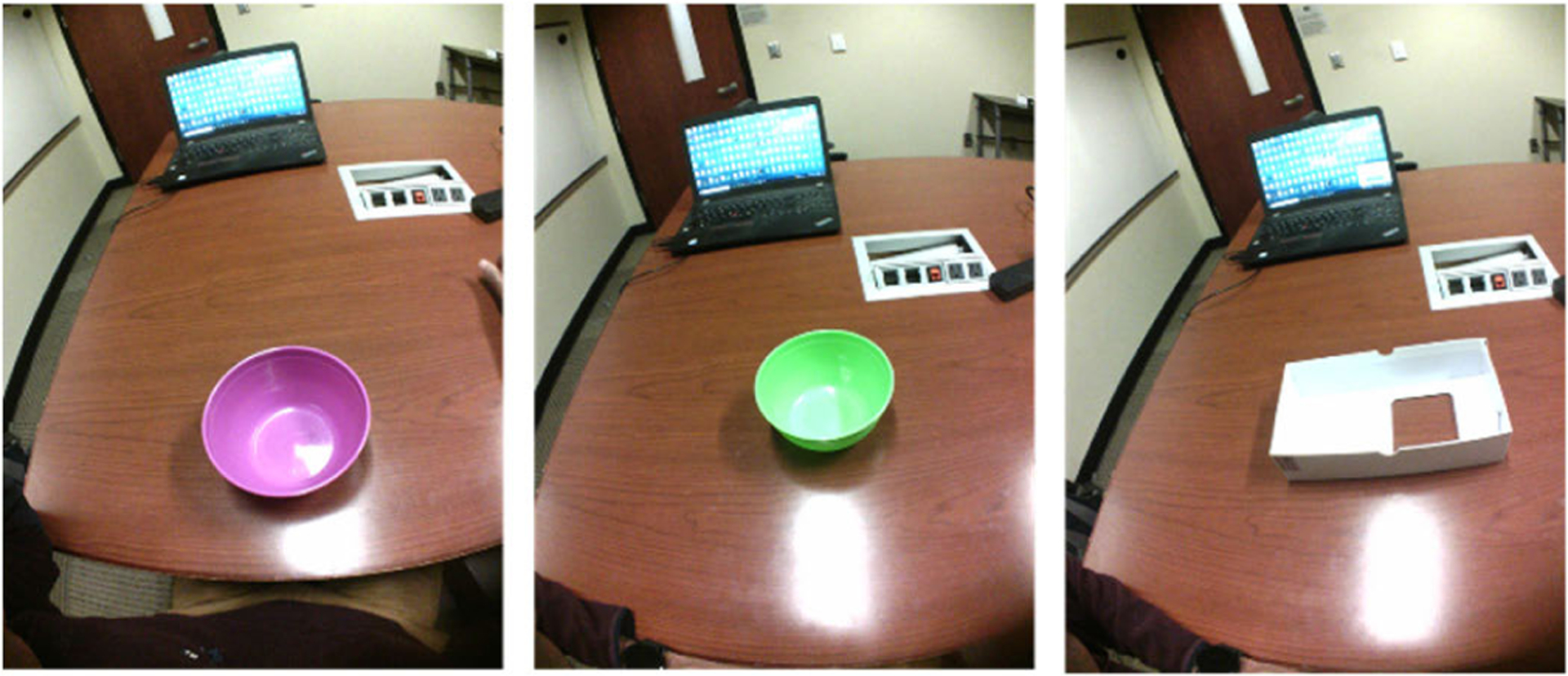
Validation images in a real-case scenario where AIM-2 was worn by a user on the eyeglass.

As a final step, four research assistants used the proposed methodology to estimate the bowl/container sizes of 3 (two circular bowls and one hollow rectangular box) shown in Fig. 8. The AIM device was worn by a user and a minimum of three images were taken for each case without any restrictions on the position/tilt of the head.

The images and the sensor signals captured by the AIM at each setup were extracted and used as input to the model. The ground-truth dimensions were measured using a tape measure.

## III. Results

**Fig. 9.**
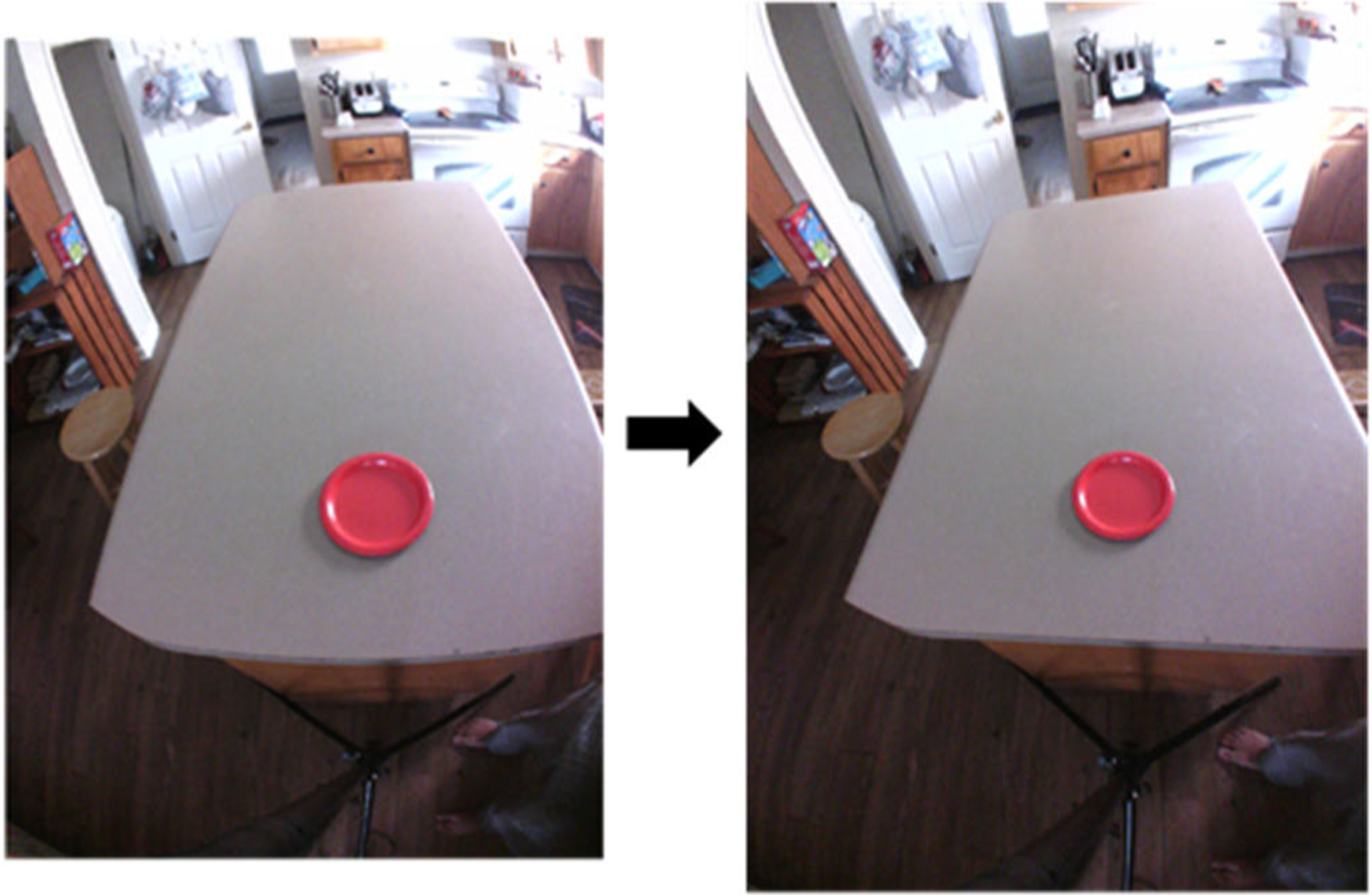
Correcting distortion due to lens aberrations [see (3)].

Fig. 9 represents a sample result of the lens corrections after (3).

**Fig. 10.**
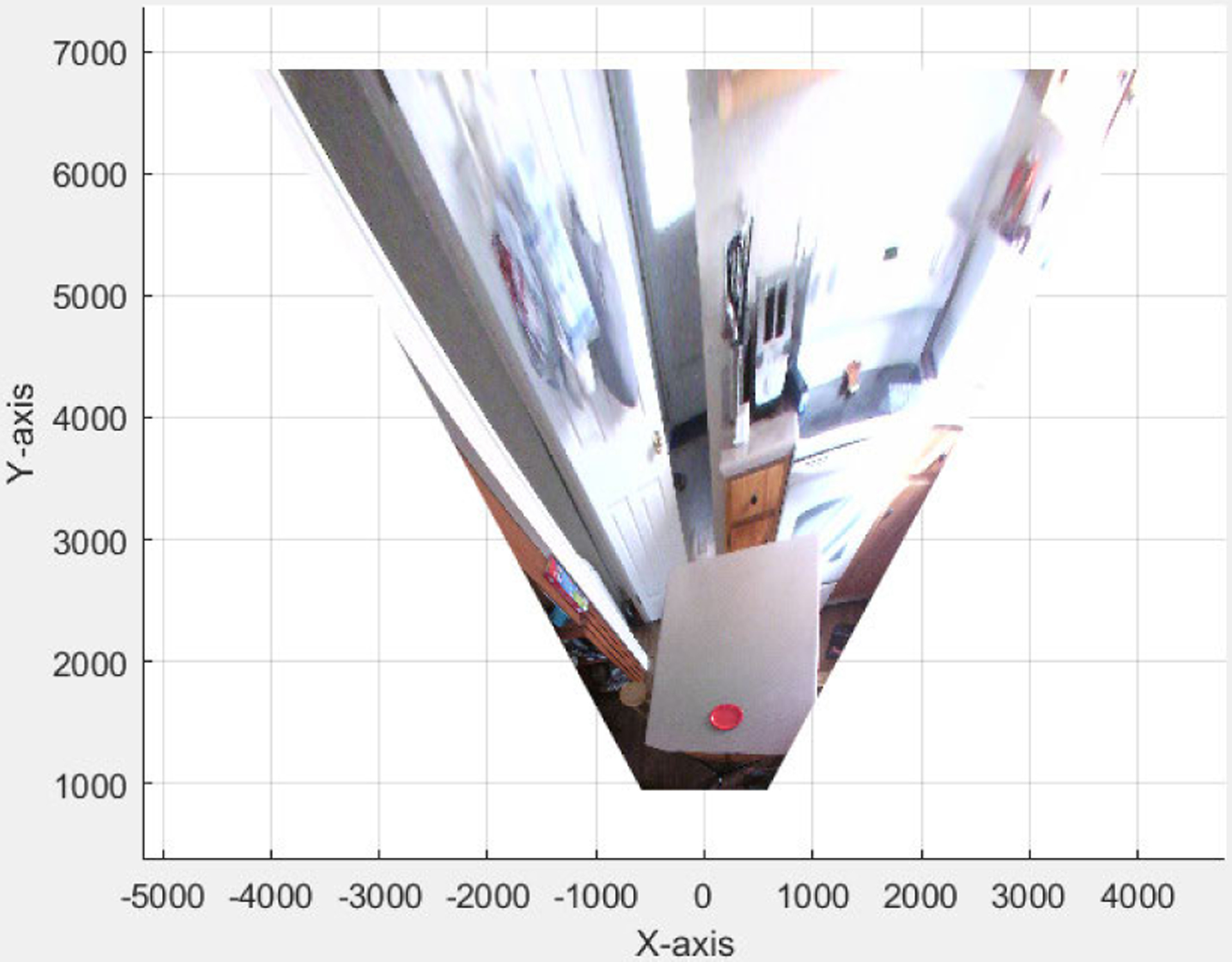
Projection of the image on the plane equation *Z* = 0 (all units are in mm).

**Fig. 11.**
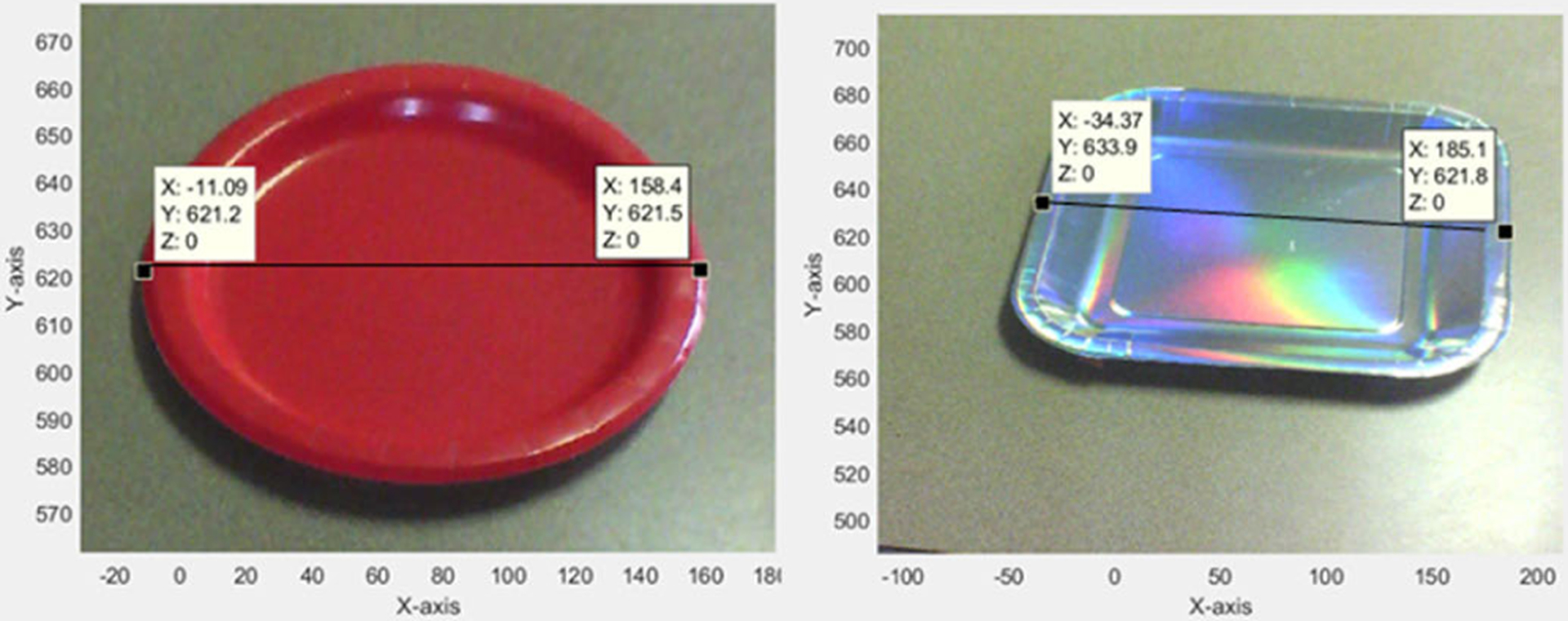
Sample measurements of plate dimensions (all units are in mm).

Using the world coordinates of the plane Z = 0 and the projected image on the plane (see Fig. 10), the dimensions of plates were measured (see Fig. 11). Any object belonging to this plane can be measured using this projection.

Table II presents the results for the dimension estimation of plates using the proposed model. The error percentage in the dimension estimation of plates was (mean ± std. dev) 2.01% ± 4.10%.

**Fig. 12.**
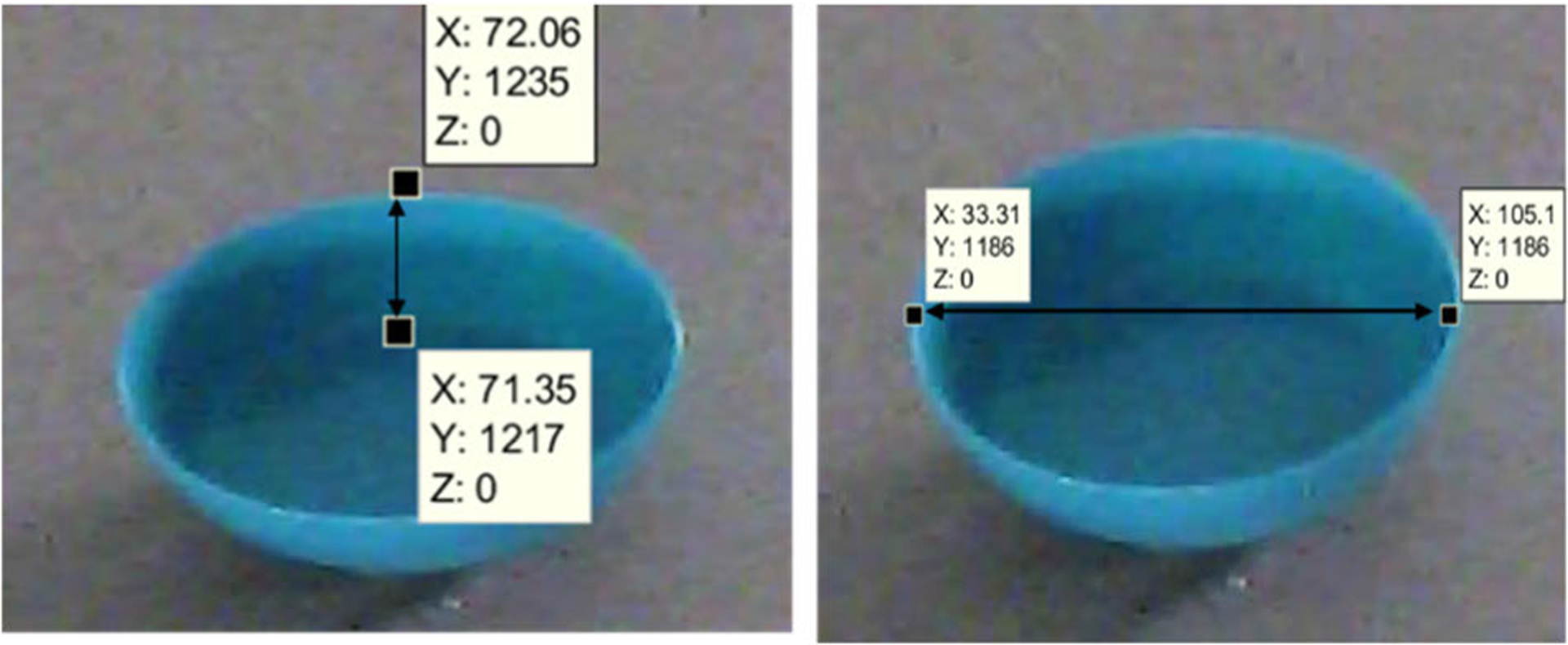
Sample measurements of bowl dimensions.

In the case of bowls, the heights of the bowls are estimated, as shown in Fig. 12. Once the height is estimated, (14) and (15) are made use of to estimate the bowl width measured at the top of the bowl (at Z = H).

Table III presents the results for the estimation of heights of bowls. The error percentage in the height estimation of bowls was (mean ± std. dev) 2.75% ± 38.11%. Table IV presents the results for the estimation of diameters of bowls. The error percentage in the diameter estimation of bowls was (mean ± std. dev) 4.58% ± 6.78%. Tables V and VI present the results from the real scenarios that were used for validation. The error percentage in the diameter/length and height estimation was, (mean ± std. dev) −7.89% ± 4.71% and 4.70% ± 11.56%, respectively.

## IV. Discussion

This study proposes a passive and automatic method for estimation of plate and bowl dimensions that involve the AIM-2 device integrated with a ToF sensor. The motivation is to use these dimensions for FPSE as in the “plate method” suggested in [17]. A geometric camera model is used to obtain real-world coordinates of the surface on which the objects of interest are present. In [1], a similar model is proposed, however, that method requires the use of a smartphone with the active participation of the user. Also, the smartphone is needed to be placed on the eating surface at a specific position. We propose a method that does not have this requirement. We make use of a ToF ranging sensor, which can directly measure the distance between the camera and the table. The method also accounts for any lens aberrations that can cause distortions such as barrel distortion in the captured images. A major contribution of this work is the elimination of fiducial markers that have been extensively used in previous methods for FPSE. The direct measurement from the range sensor will provide the necessary dimension reference in 2-D-to-3-D model conversion.

The method makes several assumptions prior to estimation: the camera axis and the range sensor axis are parallel to each other, the roll and yaw angles of the sensor are 0, the eating surface = 0, and the walls of the bowls are flat.

The proposed method was evaluated on a test bench using a calibrated protractor for positioning. Three heights and three angles were considered for testing the proposed model based on the natural behavior of participants in previous AIM-based studies. The tilt angles and the distances between the camera and the eating surface were in the selected range of pitch angles and heights.

For the measurement of bowl heights, the inner walls of the bowls were used. The rationale for using the inner walls of the bowls is that the AIM is a passive device that captures continuous images from including the start and end of meal images, and that way the images will include an empty bowl at the end of the meal. Even if the bowl is not empty, we can measure the difference between the start and end of the meal and eventually calculate the difference in the food level. This is a major advantage of having a passive camera since there are enough images covering the entire meal. It was noticed that the error rates were higher for steep angles.

The results of estimation of dimensions for plates were acceptable with good error rates. It was noticed that the dimensions were overestimated for steep angles (70°). The estimations were most accurate for 55° compared to the other two orientations. This is a promising trend since the corresponding AIM pitch angles normally occur when a person is bending forward to grab a bite of the food in front. In addition, since the AIM captures images continuously every 10 s, there will be multiple images captured at several angles due to the forward bending of the user. The angle that typically had the lowest error rates could then be picked from the range of angles available to estimate the dimensions of the objects in the scene. This reference can then also be used for images from different orientations.

We also noticed that the error rates were lower for heights of 20 and 35 cm compared to 50 cm for the same pitch angles in the case of plate diameter estimations. This could be because the plates are more central in the images as the camera is closer, reducing the field of view (the area covered by the camera). However, for the height and diameter estimations of bowls, a height 35 cm was more accurate compared to the 20- and 50-cm cases for narrow pitch angles. The 35 cm height might be ideal for the methodology used here since the walls of the bowls are clearer to the user to mark. The best results were obtained for the heights of 20 and 35 cm at 55° pitch angles for plates and bowls, respectively.

One limitation of the study is that the bowl walls are assumed to be flat and not curved. This could be a source of error in dimension measurement and portion size estimation. The method also assumes that the plates are part of the plane Z = 0. The method does not account for the thickness of the plates or the curvature of plates. However, unlike other studies which use plates as a reference, this method is not restricted to circular plates or bowls. Any shape of plates or bowls can be included.

Finally, the proposed method was validated by wearing the AIM and collecting data for three cases and four research assistants estimated the diameters/length and heights for the same. The results suggested that except for a couple of outliers (RA3: diameter and RA2: height for white box), the estimates were reasonably accurate. Also, it should be noted that one of the cases was a hollow rectangular box. This indicates that the method could be employed for similar shaped bowls and possibly for a larger variety of bowl shapes. However, in some situations where the walls of the bowls are not flat, our assumption of the walls being flat might induce errors in estimating the height of the container accurately.

Future work could include estimating food portion sizes from the dimensions of the bowls and plates. Another possible work is to use this method to estimate the dimensions of regular-shaped foods followed by food volume. Also, the proposed method was only tested on a test bench that was stationary. Since the AIM device is primarily designed to be mounted on the eyeglass, it is necessary to test the proposed method by mounting the sensor system on a human.

## V. Conclusion

In this article, we propose a wearable sensor system-based (the automatic ingestion monitor integrated with a ToF ranging sensor) method for the estimation of dimensions of plates and bowls. The contributions of this study are: 1) the model eliminates the need for fiducial markers; 2) the camera system (AIM-2) is not restricted in terms of positioning, unlike in [29] where the smartphone is required to be placed on the eating surface; 3) our model accounts for radial lens distortion in caused due to lens aberrations; 4) a distance (ToF) sensor directly gives the distance between the sensor and the eating surface; 5) the model is not restricted to circular plates; and 6) a passive method that can be used either for automatic or manual assessment of container dimensions with minimum user interaction. The error rates (mean ± std. dev) for dimension estimation were 2.01% ± 4.10% for plate widths/diameters, 2.75% ± 38.11% for bowl heights, and 4.58% ± 6.78% for bowl diameters.

## Figures and Tables

**TABLE I:** Parameters of the AIM Camera

Focal Length (f_c_)	2.4 mm
Sensor Size	3.67 mm × 2.74 mm
Image size (raw)	2592 × 1944
Image size (distortion corrected)	3378 × 2347
$c_{c}^{x}$	3378/2
$c_{c}^{y}$	2347/2
$s_{c}^{x}$	3378/3.67
$s_{c}^{y}$	2347/2.74

**TABLE II:** Error in Dimensions for Plates

Object	Height (cm)	Pitch (degrees)	Dtof (mm)	Predicted (cm)	Original (cm)	Error %	Mean Error (Mean ± Std.D)
Circular Plate (Small)	20	42	460	16.95	18	−5.84	−1.31 ± 4.82
		56	425	16.56	18	−8.00	
		64	390	19.07	18	5.94	
	35	38	740	17.13	18	−4.86	
		55	580	17.37	18	−3.48	
		72	550	18.92	18	5.11	
	50	41	870	17.61	18	−2.15	
		53	755	18.23	18	1.28	
		67	680	18.04	18	0.24	
Circular Plate (Medium)	20	42	460	20.59	22	−6.42	0.96 ± 4.66
		56	425	21.14	22	−3.93	
		64	390	23.36	22	6.17	
	35	38	740	22.51	22	2.30	
		55	580	21.47	22	−2.40	
		72	550	23.73	22	7.84	
	50	41	870	22.01	22	0.04	
		53	755	22.79	22	3.60	
		67	680	22.33	22	1.50	
Circular Plate (Large)	20	42	460	25.71	26	−1.12	5.28 ± 4.39
		56	425	25.91	26	−0.33	
		64	390	28.41	26	9.27	
	35	38	740	27.81	26	6.95	
		55	580	26.99	26	3.81	
		72	550	29.39	26	13.05	
	50	41	870	27.21	26	4.65	
		53	755	27.53	26	5.89	
		67	680	27.39	26	5.34	
Square Plate (Small)	20	42	460	17.31	18	−3.83	2.71 ± 4.38
		56	425	17.86	18	−0.80	
		64	390	19.37	18	7.62	
	35	38	740	18.33	18	1.81	
		55	580	18.34	18	1.91	
		72	550	19.88	18	10.46	
	50	41	870	18.92	18	5.10	
		53	755	18.03	18	0.18	
		67	680	18.35	18	1.93	
Square Plate (Medium)	20	42	460	21.98	23	−4.43	2.40 ± 4.73
		56	425	22.14	23	−3.72	
		64	390	24.83	23	7.96	
	35	38	740	23.93	23	4.06	
		55	580	23.09	23	0.38	
		72	550	25.18	23	9.48	
	50	41	870	24.07	23	4.64	
		53	755	23.21	23	0.90	
		67	680	23.54	23	2.35	

**TABLE III:** Error in Height Estimation in Bowls

Object	Height (cm)	Pitch (degrees)	Dtof (mm)	Predicted (cm)	Original (cm)	Error %	Mean Error (Mean ± Std.D)
Circular Bowl (Small)	20	42	460	1.96	3	−34.60	−7.40 ± 36.45
		56	425	3.04	3	1.30	
		64	390	4.44	3	48.00	
	35	38	740	1.69	3	−43.77	
		55	580	2.13	3	−29.10	
		72	550	4.31	3	43.60	
	50	41	870	1.65	3	−45.00	
		53	755	2.26	3	−24.83	
		67	680	3.53	3	17.77	
Circular Bowl (Medium)	20	42	460	6.13	7	−12.41	−12.5 ± 40.90
		56	425	7.83	7	11.81	
		64	390	1.01	7	−85.60	
	35	38	740	5.70	7	−18.64	
		55	580	5.74	7	−17.98	
		72	550	10.77	7	53.86	
	50	41	870	3.69	7	−47.29	
		53	755	5.31	7	−24.17	
		67	680	8.95	7	27.89	
Circular Bowl (Large)	20	42	460	5.17	6	−13.87	11.67 ± 36.81
		56	425	7.95	6	32.43	
		64	390	10.32	6	72.05	
	35	38	740	4.43	6	−26.17	
		55	580	5.57	6	−7.18	
		72	550	9.85	6	64.13	
	50	41	870	4.61	6	−23.22	
		53	755	5.57	6	−7.11	
		67	680	6.84	6	13.95	

**TABLE IV:** Error in Diameter Estimation in Bowls

Object	Height (cm)	Pitch (degrees)	Dtof (mm)	Predicted (cm)	Original (cm)	Error %	Error % (Mean ± Std.D)
Circular Bowl (Small)	20	42	460	6.90	7	−1.43	4.16 ± 4.38
		56	425	6.97	7	−0.43	
		64	390	7.59	7	8.43	
	35	38	740	7.02	7	0.29	
		55	580	7.55	7	7.86	
		72	550	7.79	7	11.29	
	50	41	870	7.35	7	5.00	
		53	755	7.18	7	2.57	
		67	680	7.27	7	3.86	
Circular Bowl (Medium)	20	42	460	10.28	11	−6.55	2.52 ± 7.66
		56	425	10.26	11	−6.73	
		64	390	11.94	11	8.55	
	35	38	740	10.26	11	−6.73	
		55	580	11.64	11	5.82	
		72	550	12.66	11	15.09	
	50	41	870	11.53	11	4.82	
		53	755	11.49	11	4.45	
		67	680	11.44	11	4.00	
Circular Bowl (Large)	20	42	460	14.62	15	−2.53	7.04 ± 7.73
		56	425	14.41	15	−3.93	
		64	390	17.44	15	16.27	
	35	38	740	15.11	15	0.73	
		55	580	16.92	15	12.80	
		72	550	17.55	15	17.00	
	50	41	870	15.97	15	6.47	
		53	755	16.00	15	6.67	
		67	680	16.49	15	9.93	

**TABLE V:** Error in Diameter Estimation in Containers

Object	Predicted Diameter (cm)					Ground Truth Diameter/Length (cm)	Error (%)
	RA1	RA2	RA3	RA4	Mean		
White Box	25.70	25.18	7.17	26.37	21.11	24.50	−13.85
Pink bowl	15.10	15.75	15.10	15.39	15.33	14.20	−7.99
Green Bowl	14.83	15.20	15.41	15.25	15.17	14.90	−1.83

**TABLE VI:** Error in Height Estimation in Containers

Object	Predicted Height (cm)					Ground Truth Height (cm)	Error (%)
	RA1	RA2	RA3	RA4	Mean		
White Box	3.46	1.563	5.66	2.98	3.42	3.8	−10.11
Pink bowl	6.185	9.62	6.7	5.841	7.09	6	18.11
Green Bowl	5.792	8.3	8.06	7.556	7.43	7	6.10
